# Idiopathic Hypersomnia Patients Revealed Longer Circadian Period Length in Peripheral Skin Fibroblasts

**DOI:** 10.3389/fneur.2018.00424

**Published:** 2018-06-07

**Authors:** Linus Materna, Hartmut Halfter, Anna Heidbreder, Matthias Boentert, Julian Lippert, Raphael Koch, Peter Young

**Affiliations:** ^1^Division of Sleep Medicine and Neuromuscular Disorders, Department of Neurology, University Hospital Muenster, Muenster, Germany; ^2^Department of Neurology, Inselspital, University Hospital Bern, University of Bern, Bern, Switzerland; ^3^Institute of Biostatistics and Clinical Research, University of Muenster, Muenster, Germany

**Keywords:** idiopathic hypersomnia, circadian rhythm, circadian period length, clock genes, fibroblasts

## Abstract

The vast majority of living organisms have evolved a circadian rhythm of roughly 24 h in adaptation to ever-changing environmental conditions, such as the cycle of light and darkness. In some sleep disorders like idiopathic hypersomnia (IH) this adaptation is defective. As the etiology of this disease is largely unknown, we examined the *in vitro* circadian period length of patients suffering from IH. The patients were diagnosed according to the ICSD3-criteria by clinical history, polysomnography (PSG), and multiple sleep latency testing (MSLT). In order to gain insight into the molecular mechanism of this sleep disorder we collected fibroblasts from skin biopsies of IH patients and healthy subjects. We determined the circadian period length of the primary fibroblast cells by lentiviral infection with a construct expressing a luciferase gene under the control of a *BMAL1* promoter. The group of IH patients revealed on average a prolonged circadian period length. In comparison to the group of healthy controls (HC) the mean period length was estimated to be 0.82 h (95%-CI 0.44–1.20 h) longer in the patient group. This finding further stresses a disturbed regulation of the circadian rhythm in IH patients as part of the pathophysiology of this complex and poorly understood primary sleep disorder.

## Introduction

According to the recent classification of sleep disorders, central disorders of hypersomnolence are subdivided into different categories including narcolepsy with cataplexy (NT1), narcolepsy without cataplexy (NT2), recurrent hypersomnia (Kleine-Levin-Syndrome, KLS), and idiopathic hypersomnia (IH) (1). The key manifestation in all categories is excessive daytime sleepiness. IH is a primary sleep disorder of not yet finally resolved etiology characterized by a high and disabling amount of sleep required during the day, despite normal or even prolonged sleep times at night. It is a rare disease with an estimated prevalence of 0.5/100.000 people (2, 3). No current reliable epidemiological data are available due to a lack of any clinical observational studies. The symptoms of the disease include extreme difficulties waking up, excessive daytime sleepiness (EDS) with extensive, mostly involuntary and unrefreshing daytime naps in addition to problems with concentration and prominent mood disturbances. Sleep drunkenness, sleep inertia, and hypnagogic hallucinations are also frequently reported (4).

Disease onset occurs most often during adolescence or young adulthood and is accompanied by severe social and economic impairments for the patients, resulting in a great loss in the patient's quality of life. Currently there is no admitted medical treatment for IH. However, a recent double-blind, placebo-controlled study suggests Modafinil might be efficacious in treating IH patients (5).

Sleep regulation seems to be disturbed in patients suffering from IH (6, 7). This might be due to a pathologically high sleep pressure, a lack of an adequate homeostatic antagonist or the malfunctioning of the underlying circadian process. Reports of distinctive HLA markers have been inconsistent (8). A series of 138 patients with IH found that the prevalence of inflammatory disorders, allergies, and the incidence of family members with inflammatory disorders was increased in patients compared with controls (9).

Concerning the pathophysiology of IH, one study suggested that an un-specified endogenous substance in the cerebrospinal fluid of some patients with primary hypersomnia, including IH, might enhance inhibitory signaling through gamma-aminobutyric acid type A (GABA_A_) receptors, thereby promoting sleepiness. The effects were reversed *in vitro* by flumazenil, which also normalized vigilance in seven patients *in vivo* (10). A pilot study from the same group found that clarithromycin as a GABA_A_ affecting drug improved subjective sleepiness without improving objective signs of alertness in patients with evidence of abnormal GABA_A_ potentiation (11). In contrast, a separate study could not confirm *in vitro* GABA_A_ potentiation with cerebrospinal fluid from 15 patients with IH (12). Hypocretin-1, the wake-promoting neurotransmitter that is absent or deficient in patients with narcolepsy type 1, is normal in patients with IH (13, 14). Findings about the contribution of the monoaminergic transmitter system and central acting substances are inconsistent. Therefore a malfunctioning of the underlying circadian system should be considered as an alternative cause of IH.

Numerous autonomous circadian clocks can be found in nearly all different tissues of the body, coordinated by a central master clock located in the hypothalamus–the suprachiasmatic nucleus (SCN). The SCN integrates the information about exogenous factors such as light and then orchestrates the activity of the circadian oscillators in peripheral tissues via systemic and endocrine signals. Thereby the SCN adjusts the physiological processes by which the organism responds to and anticipates the diurnal requirements and thus resets the internal clock every day. This process is called “entrainment.”

Within the cell, the molecular mechanism underlying the course of the circadian rhythms has been identified as a feedback mechanism that involves transcriptional, translational and post-translational cycles of clock genes and their products (15). At the transcriptional level a complex mechanism of interconnected transcriptional regulation leads to the diurnal alternating expression of clock-controlled genes (*ccg*) and transcriptional regulators such as *BMAL1, CRY1* and *-2*, and *PER1*,−*2* and *-3* (16), generating the circadian rhythm of the cells. The duration of this rhythm is therefore genetically determined and can be measured as the circadian period length (τ). In the healthy population the circadian period length is on average τ = 24.3 h (17), which is slightly longer than the duration of the actual day, making the process of entrainment necessary.

The length of the circadian period of the cells can be monitored using a lentiviral bioluminescence assay (18, 19). It has been demonstrated that fibroblasts from subjects with different chronotypes displayed different circadian period lengths (20, 21), indicating a connection between the daytime preference or diurnal pattern of activity and the endogenous circadian period of the subject.

We have recently shown that the mRNA expression of several clock genes was impaired in fibroblast cells from IH patients compared to healthy controls (HC) (22). We hypothesized that the excessive sleepiness seen in IH patients partly results from an altered circadian rhythm. To answer this, we investigated the circadian period length in isolated dermal fibroblast cells from patients suffering from IH and compared them to cells from healthy volunteers to gain further insight into the sleep and wake regulation at the molecular level.

## Materials and methods

### Study patients and controls

All procedures, consent forms and questionnaires were approved by the Medical Ethical Committee of the University Muenster (ref.-no.: 2009-361-f-S, renewed 08/05/2017). All patients and controls gave signed written informed consent.

Fifteen patients suffering from IH were consecutively recruited from the Department of Neurology, Division of Sleep Medicine and Neuromuscular Diseases at the University Hospital Muenster. Diagnosis was made according to standard criteria of the ICSD-3 (1) as described recently (23). The Epworth Sleepiness Score (ESS) to assess subjective daytime sleepiness (24) had to be above 10 and the Horne-Ostberg (HO) score was evaluated by the self-assessment HO questionnaire (25). All patients underwent polysomnography (PSG) for at least one night to prove high sleep efficiency, followed by a Multiple Sleep Latency Test (MSLT). MSLT had to be below 8 min on average with less than 2 Sleep Onset REM Episodes (SOREM), so no 24 h PSG had to be conducted. Sleep efficiency had to be above 85%. Patients with comorbid psychiatric or neurological disorders, as well as other disorders explaining EDS including obesity (BMI > 35 kg/m^2^) were excluded. Cerebrospinal fluid (CSF) investigation was performed in all patients to exclude other central causes for sleepiness (for example, chronic inflammatory disorders) and to exclude hypocretin deficiency.

Sixteen HC, unrelated to the patients, were included.

### Cell culture of human fibroblasts

Establishing of fibroblast cell cultures was performed as described previously (22). Briefly, a 2 mm punch biopsy was taken from the lateral pelvic area. The wound was dressed and the biopsy split into halves. Each half was taken into culture on a 35 mm dish to gain two biological replicates and supplied with 1 ml Dulbecco's modified Eagle medium with 20% fetal calf serum (FCS), 1% Penicillin-Streptomycin mixture, and 2 mM Glutamax (all obtained from Life Technologies, Netherlands) and incubated at 37°C and 5% CO2. As soon as the cells reached confluence, the newly grown fibroblasts were harvested.

### Lentivirus production and titration

The lentiviral vector was generated by co-transfection of plasmids pMD2.G and psPAX2 together with a reporter construct (pLV7Bmal) into HEK293FT cells as described previously (26), producing a lentiviral construct expressing the luciferase gene under the control of a mouse *Bmal1* promoter. HEK293T cells were cultivated in 100 mm dishes in a medium containing Dulbecco's modified Eagle medium (DMEM) containing 2 mM Glutamax (Sigma-Aldrich, Germany), 10% fetal calf serum (FCS; Thermo Fisher Scientific), 1% penicillin-streptomycin (Sigma-Aldrich), and 1 mM sodium pyruvate (Sigma-Aldrich) at 37°C and 5% CO2. Each dish was incubated with a mix of pMD2.G, psPAX2, and pLV7Bmal in OptiMEM (Life Technologies, Netherlands) containing transfection reagent polyethylenimin (PEI, Polysciences, 1 mg/ml) at 20°C for 30 min. The transfection reagent-DNA mixture was then added to the cells and incubated for 20 h at 37°C and 5% CO2 before the medium was replaced by 12.5 ml medium supplemented with 1 M hydroxyethyl piperazineethanesulfonic acid (HEPES; 25 mM final concentration). At 48 h post-transfection, the lentivirus-containing medium was collected, filtered through 0.45 μm filters and centrifugation at 4,000 *g* for 10 min to eliminate the cells and cell debris. The transfected HEK cells were supplied with 12.5 ml fresh medium with 25 mM HEPES as described before and a second supernatant was collected after another incubation for 24 h. Lentivirus particles were concentrated by centrifugation at 27,000 g for 5 h at 20°C, re-suspended in 650 μl of PBS and used immediately for infection of fibroblast cells.

### Fibroblast infection and evaluation of circadian rhythms

Fibroblasts were seeded on 96-well dishes for infection in at least triplicates per cell line. The next day the cells were infected by the addition of lentivirus particles re-suspended in fibroblast medium with 10% FCS including 8 μg/ml polybrene (Sigma-Aldrich). Twenty-four hour post-infection, the cells were selected with DMEM, 10% FCS and 10 μg/ml blasticidin. After 4–5 days, the fibroblasts were synchronized by addition of dexamethasone (Sigma-Aldrich) to a final concentration of 100 nM for 2 h. After rinsing the cells with PBS, recording medium (DMEM without phenol red (Thermo Fisher), including Glutamax and 25 mM HEPES buffer pH 7.4 (Capricorn, Germany), 10% FCS, and 1% PNS) supplemented with 0.1 mM beetle luciferin (Promega, Heidelberg, Germany) was added to each well. Bioluminescence was recorded using a luminescence reader (Biotek, Germany) directly within the culture incubator at 37°C as described previously (27). The measurements were performed at an interval of 14 min for 6–7 d and data were recorded by the use of Gen5 software (Biotek, Germany).

The analysis was performed by the use of MultiCycle® Analysis software (Actimetrics). An interval of 8 to 60 h was selected for the calculation of the period length. The circadian period length was computed with MultiCycle®, which identifies the period length of a sinusoidal component. It employs a gradient descent method to find a least squares fit to a dampening sine wave.

All fibroblasts lines were successively measured in different runs and differences between the period length of IH patient's fibroblasts and HC were calculated on measurement level.

### Statistical analysis

Standard descriptive analyses were performed. Categorical variables are shown as absolute and relative frequencies and continuous variables are presented as mean ± standard deviation or median [25%-quantile (Q25)−75%-quantile (Q75)]. Fisher's exact test was calculated to compare sex between HC and patients and Mann-Whitney *U* tests were performed for age, Epworth Sleepiness Score, and Horne-Ostberg Score.

First, in the univariate analysis of the period length, all samples from one patient were regarded as independent. Secondly, the period lengths in patients and controls were estimated within a linear mixed model, which accounted for dependencies between multiple samples within a patient and within a single run, and for varying number of samples per patient (missing values were treated as missing at random). The dependent variable was the period length (h) which was calculated as the mean of at least three replicates within one multiwell plate. The fixed factor was group (patient/control). The model was fitted including a random intercept for each patient and a random intercept for each run to take the clustered data structure into account. Both intercepts were modeled as uncorrelated. Results are reported as mean estimates or regression coefficients and corresponding 95% confidence intervals (95% CI) and *p*-values from the Wald tests. Within the mixed model, intraclass correlation coefficients (ICCs), representing the ratio of the between-cluster variance to the total variance, were calculated for measurements within one patient and for samples within one multiwell plate. The ICCs can be interpreted as the proportion of the total variance in the period length that is accounted for by the clustering of run and patient or as the correlation among observations within the same cluster.

To analyze the correlation between the period length and the Epworth sleepiness score and Horne-Ostberg score, the mean period length over all samples and measurements was calculated on the subject level. Then, Spearman's rank correlation coefficients were calculated.

Statistical analyses were performed using SAS software, version 9.4 of the SAS System for Windows (SAS Institute, Cary, NC, USA). Inferential statistics like *p*-values and confidence intervals were intended to be exploratory, not confirmatory. Therefore, neither global nor local significance levels were determined, and no adjustment for multiplicity was applied. *P*-values ≤ 0.05 were considered as statistically noticeable.

## Results

For the analysis of the circadian period length, 15 patients with IH (80% female, *N* = 12) and 16 HC (56% female, *N* = 9), with a mean age of 33.5 ± 10.1 (IH) and 30.7 ± 13.3 (HC), were included in the study (Table 1). The data were obtained in our sleep laboratory and from self-assessed questionnaires.

**Table 1:** Clinical characteristics of the study patients and healthy controls.

**Clinical features**	**Idiopathic hypersomniacs**	**Controls**	***P*-values**
Frequency	*N* = 15	*N* = 16	
Age	33.5 ± 10.1 (*N* = 15) 32 (25–42)	30.7 ± 13.3 (*N* = 16) 27 (23–30)	*p* = 0.321^a^
Females %	80 (12 from 15)	56 (9 from 16)	*p* = 0.252^b^
Epworth sleepiness score	15.8 ± 3.1 (*N* = 14) 16 (14–18)	5.1 ± 2.1 (*N* = 16) 5.5 (4–6)	*p* < 0.001^a^
Horne-Ostberg score	43.3 ± 11.5 (*N* = 7) 44 (33–56)	53.2 ± 11.3 (*N* = 16) 53 (49–60.5)	*p* = 0.09^a^
MSLT, min	5.0 ± 2.2 (*N* = 15) 5.0 (4.0–6.9)		
PSG time in bed, min	459.4 ± 38.7 (*N* = 15) 467 (442–477.5)		
PSG total sleep time, min	427.9 ± 39.9 (*N* = 15) 432 (399–438)		
PSG sleep efficiency%	93.1 ± 4.4 (*N* = 15) 95 (90.7–95.3)		
REM sleep%	20.1 ± 5.4 (*N* = 15) 20.6 (16.4–21.5)		
N2%	48.7 ± 6.4 (*N* = 13) 47.3 (45.5–50.0)		
N3 %	24.1 ± 6.0 (*N* = 15) 23.4 (20.7–29.8)		
Periodic leg movements, n/h	2.8 ± 5.0 (*N* = 15) 0.7 (0.0–2.8)		
Apnea/hypopnea, n/h	1.9 ± 3.6 (*N* = 15) 0.7 (0.1–2.0)		
Qxygen desaturation, n/h	1.1 ± 2.0 (*N* = 14) 0.3 (0.0–1.4)		
Hemoglobin g/dl	13.7 ± 1.1 (*N* = 15) 13.4 (13.0–14.8)		
TSH mU/l	1.5 ± 0.7 (*N* = 15) 1.3 (0.9–2.0)		

Age, age at diagnosis/biopsy; MSLT, multiple sleep latency test; PSG, Polysomnography, which was performed from about 10:30 p.m. to 6:00 a.m. (450 min); REM, rapid eye movement; N2, sleep stage 2; N3, sleep stage 3/slow wave sleep; TSH, thyroid stimulating hormone. Variables are reported as absolute and relative frequencies, mean ± standard deviation or median (25–75%-quantile). P-values are from

a Mann-Whitney U test and

b *Fisher's exact test*.

**Figure 1:**
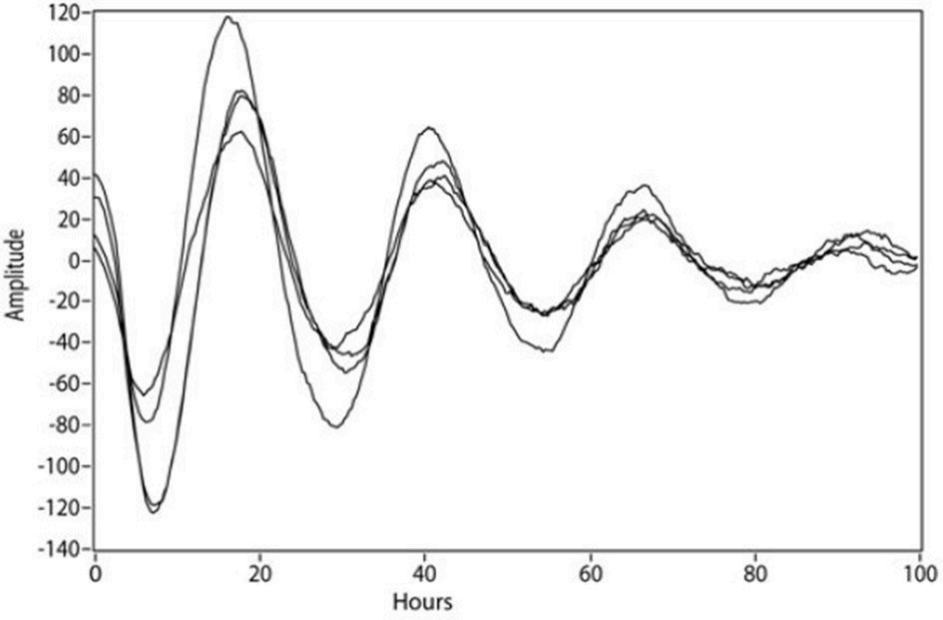
Luminescence detected from human fibroblasts reflecting the circadian expression of a luciferase gene under control of the BMAL1 promoter.

Performing a lentiviral bioluminescence assay on skin fibroblasts, the rhythmic expression of the Bmal1-luciferase construct revealed a harmonic circadian rhythm over a time period of up to 100 h (Figure 1). We found a circadian period length with a median value of 25.47 h (Q25: 24.87–Q75: 26.08 h) for the IH patients if all samples were regarded as independent. In contrast the group of HC revealed a period length with a median value of 24.53 h (Q25: 24.15–Q75: 25.32 h) with a difference of 0.94 h (56 min) between the median values (*p* < 0.001).

**Figure 2:**
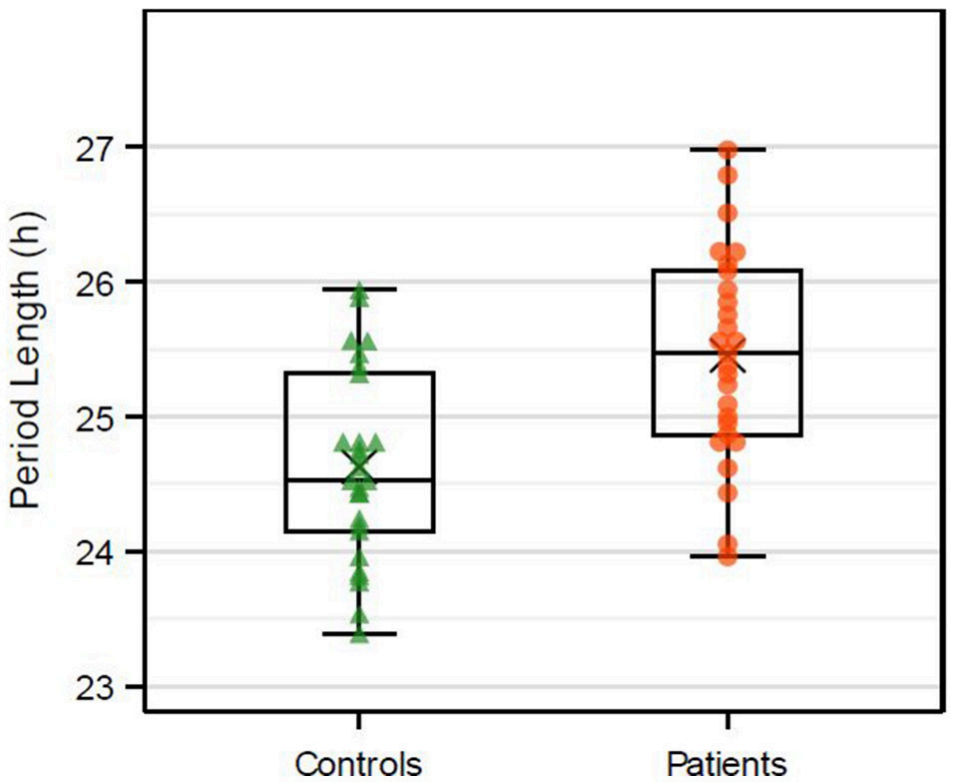
Boxplots of the *in vitro* period length (h). Dots represent the mean of at least three replicates on three different wells within one plate. Multiple dots per patient are included. X represents the mean value.

In the mixed model analysis the adjusted estimated mean period length was 0.82 h (95% CI 0.44–1.20 h, *p* < 0.001; Figure 2) longer in patients than in healthy control subjects. Estimated mean period length in patients was 25.3 h (95% CI 24.7–25.9 h) and in healthy subjects 24.5 h (95% CI 23.9–25.1 h). The intra-class correlation coefficient representing the proportion of total variance of the period length explained by cluster variable was ICC = 0.393 for measurements within a run and for the patient cluster ICC = 0.066.

**Figure 3:**
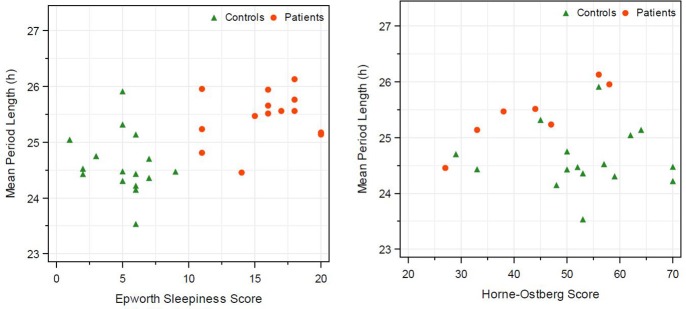
Correlation of *in vitro* period length and Epworth sleeping score and *in vitro* period length with morning-eveningness score (Horne Ostberg). Healthy subjects are represented by triangle and IH patients by circles. Calculation of Spearman correlation is given in Table 2.

Correlation between the *in vitro* period length and ESS was weak. In both groups, IH and HC, no correlation between *in vitro* period length and ESS was observed as indicated by Spearman correlation (Figure 3). Concerning the correlation of *in vitro* periods and HO score (indicating the chronotype of the subjects), a high Spearman correlation coefficient was observed within the IH patients (0.86, *N* = 7). No correlation was observed in the healthy control group (−0.01, *N* = 16) and pooled over both groups (−0.08, *N* = 23; Table 2).

**Table 2:** Spearman correlation coefficients between sleeping scores (Epworth sleeping, Horne-Ostberg) and *in vitro* period length.

	**Period length (h)**		
	**Overall**	**Controls**	**IH Patients**
HO-score	−0.08 (*N* = 23)	−0.01 (*N* = 16)	0.85 (*N* = 7)
ESS-score	0.54 (*N* = 30)	−0.34 (*N* = 16)	0.13 (*N* = 14)

## Discussion

The etiology of IH is not well understood, but it is likely to have a genetic component (28). The present study focuses on the endogenous human circadian rhythm as a contributing factor to the emergence of IH. Here, we investigated the genetically encoded period length of the circadian clock in fibroblast cultures which is cell-autonomous. We measured this property of the cells under constant conditions to exclude the influence of most exogenous factors like inflammatory parameters or the monoaminergic system on the circadian rhythm. The fibroblasts of IH patients revealed a prolonged circadian period length in comparison to HC, providing further evidence that clock genes and their specific action in modulating the circadian rhythm might be involved in the pathophysiology of IH. These results are in concordance with the finding of a phase delay in the rhythm of melatonin and cortisol secretions in 15 patients with IH (29). Clinical findings like sleep inertia and sleep drunkenness, which are often reported by IH patients, indicate an altered homeostatic regulation of the sleep wake cycle of the patients (30), which might also be linked to an aberrant circadian rhythm and a long biological night (31). Focusing on the endogenous circadian regulation, this study shows for the first time a prolonged circadian period length in fibroblasts of patients suffering from IH.

Fibroblasts are highly suitable for the study of circadian rhythms as they reflect the circadian rhythm of the central oscillator, the SCN (32). Several publications have demonstrated a correlation between chronotypes and the measurement of period length *in vitro* (18, 28, 29). Fibroblasts also replicate the behavioral changes that appeared as a consequence of mutations in clock genes of humans suffering from a familial advanced sleep phase syndrome (FASPS) (33, 34). Pagani et al. (35) have shown that the period length determined *in vitro* correlates with the values obtained by *in vivo* measurements including melatonin secretion and sleep behavior under laboratory conditions. Therefore, measuring circadian rhythm in fibroblasts of IH patients was a further step in elucidating the underlying molecular mechanisms of this disease.

On average the IH patient revealed a prolonged period length. A behavioral output of this longer rhythm is supposed to be a late chronotype, as demonstrated for a healthy population (20). Low HO scores correlate with a late chronotype and on average the IH patients in our study had a lower HO score by 9.9 points [IH: 43.3 (*N* = 7) vs. HC: 53.2 (*N* = 16)], but a larger cohort needs to be examined to validate this finding. The IH patients showed a relatively strong positive correlation between period length and HO score (Figure 3), which seems to be in contrast to the formerly mentioned relation of period length and HO score, but might also be due to a random effect of this small cohort. In the control subjects we found no correlation between HO score and the *in vitro* period length (Figure 3), which is consistent with the findings of Hasan et al. (36).

A prolonged *in vitro* period was also observed in a similar approach using fibroblasts of patients with a non-24-h-sleep-wake rhythm disorder (N24SWD). This circadian disorder is characterized by a delay in daily sleep timing of 30 to 60 min each day (37), leading to a desynchronization of the internal and external rhythm and subsequently recurring episodes of excessive daytime sleepiness. These authors reported that fibroblasts of patients with a delayed sleep-wake phase disorder (DSWPD) did not show an abnormal period length, indicating that other factors than the circadian period length must be fundamental in this disease. A defective response to the Zeitgeber light might be the cause of this disease, leading to a delayed cortisol peak in the blood and a delayed reset of the circadian rhythm, while the molecular mechanisms in the cells does not have to be affected. This is supported by the fact that DSPS patients do not suffer from excessive daytime sleepiness and a reduced level of alertness as long as they are allowed to follow their individual sleep pattern. In free-running disorder N24SWD, often found in individuals with complete blindness, a prolonged circadian period leads to complications due to a malfunctioning of the entrainment process (38). However, a recent study on circadian period length of blind subjects demonstrated that their fibroblasts revealed the same period length while their physiological period length was longer compared to healthy subjects (35). Other processes downstream of light perception lead to the entrainment of the cells throughout the body. Nevsimalova et al. (29) showed a significant delay in melatonin and cortisol secretion in 15 IH patients, indicating a disturbance in the entrainment (29). Soluble serum factors, for example, can affect the period length of the circadian rhythm as it has been shown in experiments with cells and serum from older vs. younger subjects (39). The shorter behavioral circadian rhythm of elderly subjects was not reflected *in vitro* by a shorter period length in fibroblast cell cultures, meaning that the endogenous rhythm of the cells does not change in the process of aging. Soluble serum factor(s) though, obtained from serum of older subjects led to a shorter rhythm in fibroblast cells from younger and older subjects *in vitro*, while the serum of younger subjects lacked such a factor (39). An altered susceptibility to these currently un-identified factors could also contribute to the different behavioral rhythms seen in IH patients. However, the influence of exogenous factors on the observations of the present study can be ruled out due to identical incubation conditions.

Several studies argue for the contribution of genetic factors, which is found in approximately 30 to 40 percent of their study patients (3, 28, 40). Whether the prolonged circadian rhythm found in our study results from genetic aberrations remains to be shown. Apart from genetic factors, an enhanced activity of a GABA_A_-receptor stimulating-factor could be detected *in vitro* in the CSF of IH patients that could also contribute to the pathophysiological mechanism of these patients (10). This depicts a possible mechanism leading to IH, but as GABA is a neurotransmitter it is not likely to interfere in the regulation of the fibroblast cell function. Moreover we conducted our experiments without the influence of GABA and the unspecified agent(s) contained in the CSF. Therefore our finding stands separately from the central nervous strain and displays a different part of the pathophysiology of IH.

Our study bears some limitations. All inference statistics have only an exploratory interpretation. The multivariable models were fitted data-driven and were not determined before data collection. Therefore, the applied statistical models are not valid to make assumptions about population inference. Due to the sample size, fitting of larger multivariable models with further clinical covariables was not possible. Consequently, prospective studies are required to confirm the difference of the period length between patients and controls. In the clinical setting of our sleep laboratory the 24-h PSG and actigraphy is not performed as standard method. Sleep deprivation was excluded by sleep diaries and detailed anamnesis. A further limitation is the fact that sex was not matched between IH patients and control subjects. However, the results from the study of Pagani et al. (35) show that there was no difference between one group of their subjects with a high percentage of females for the *in vitro* period length compared to the other groups with a lower proportion of females.

We have identified here that the clinical phenotype of IH with its excessive daytime sleepiness is associated with a prolonged circadian period length in skin fibroblasts compared to healthy subjects. In future studies the *in vivo* period length of IH patients should be measured, by analyzing the free-running period under constant dim light conditions for example, and directly correlated with the data of the *in vitro* period length, to strengthen our results. With the finding of this study we suggest that the prolonged circadian period length found in IH patients is a crucial part in the etiology of IH and we hope that future investigations will further focus on the circadian dysregulation and the underlying molecular processes resulting in IH.

## Author contributions

LM: performing experiments, analysis, and interpretation of data, writing manuscript; HH: planning and performing experiments, analysis and interpretation of data, writing manuscript; AH: acquisition of patients and revising the manuscript; JL: acquisition of patients and revising the manuscript; MB: acquisition of patients and revising the manuscript; RK: performing statistical analysis and interpretation of data, writing the manuscript; PY: study supervision and revising the manuscript.

### Conflict of interest statement

The authors declare that the research was conducted in the absence of any commercial or financial relationships that could be construed as a potential conflict of interest.
